# Antioxidant Activity and Spectroscopic Characteristics of Extractable and Non-Extractable Phenolics from *Terminalia sericea* Burch. ex DC.

**DOI:** 10.3390/molecules23061303

**Published:** 2018-05-29

**Authors:** Chinedu Anokwuru, Muendi Sigidi, Marlaine Boukandou, Peter Tshisikhawe, Afsatou Traore, Natasha Potgieter

**Affiliations:** 1 Chemistry Department, University of Venda, Private Bag X5050, 0950 Thohoyandou, South Africa; anokwuruchi@gmail.com; 2 Microbiology Department, University of Venda, Private Bag X5050, 0950 Thohoyandou, South Africa; Muedi.sigidi@univen.ac.za (M.S.); bouk_marlaine@yahoo.fr (M.B.); 3 Botany Department, University of Venda, Private Bag X5050, 0950 Thohoyandou, South Africa; peter.tshisikhawe@univen.ac.za; 4 School of Mathematical and Natural Sciences, University of Venda, Private Bag X5050, 0950 Thohoyandou, South Africa; natasha.potgieter@univen.ac.za

**Keywords:** extractable, non-extractable, antioxidant activity, agglomerative hierarchical clustering, principal component analysis, multivariate analysis, nuclear magnetic spectroscopy

## Abstract

The aim of this study was to determine the antioxidant activity of the extractable and non-extractable phenolics of *Terminalia. Sericea* Burch. Ex DC. Free, ester bound, ether or glycoside bound and insoluble phenolics were extracted from the fruit, leaves, stem, and root samples. Follin Ciocalteu was used to estimate the phenolic content while DPPH (2,2-diphenyl-1-picrylhydrazyl) assay was used to determine the antioxidant activity. The data obtained were subjected to multivariate analysis for relationships. The result indicated that the highest average total phenolic contents and antioxidant activities were found in the free (14.8 mgGAE/g; IC_50_ 6.8 μg/mL) and ester bound (15.1 mgGAE/g; IC_50_ 6.4 μg/mL) extractable phenolics. There was a strong negative correlation between TPC and DPPH (r = −0.828). Agglomerative hierarchical clustering revealed three clusters. Cluster one contained the insoluble and glycoside phenolics while cluster 2 contained only free phenolic acid of the root. The third cluster was predominantly free and ester bound phenolic extracts. The principal component analysis score plot indicated two major clusters with factor 1 (F1) explaining 61% of the variation. The nuclear magnetic resonance spectroscopy spectra indicated that gallic acid and resveratrol are the major phenolic compounds present in the root. This study has demonstrated that extractable phenolics contributed more to the antioxidant activities compared to the non-extractables.

## 1. Introduction

Accumulation of reactive oxygen and nitrogen species during oxidative metabolic processes can result in diseases such as diabetes, aging, inflammation, cardiovascular, neurodegenerative, and brain dysfunction [1,2,3]. Antioxidants are molecules that prevent oxidative damage caused by reactive species through scavenging free radicals, inhibiting lipid perodixation, and metal chelation [1,4]. Synthetic antioxidants such as butylatedhydroxytoluene (BHT), butylatedhydroxyanisole (BHA), and propylgallate (PG) are commercially used to preserve food by increasing the shelf-life through the inhibition of lipid peroxidation [4,5,6]. However, studies have revealed that synthetic antioxidants are toxic and carcinogenic, leading to the search for alternative sources of antioxidants from the natural origin [7]. 

Food and medicinal plants have been identified as rich natural sources of antioxidants [8]. Phenolics are secondary metabolites in plants and are known as major sources of antioxidants [9,10]. They protect plants against UV radiation, oxidative stress, and microbial infections [11,12,13,14]. The health benefits of phenolics have been ascribed to their pharmacological potentials such as anti-carcinogenic, anti-inflammatory, and anti-microbial [15]. Phenolics are good antioxidants because of their ability to scavenge free radicals, reactive oxygen and nitrogen species, and metal chelation [16]. They are generally classified as phenols, polyphenols, stilbenes, xanthones, and coumarins [17]. Phenolic acids are further classified as hydroxybenzoic acid and hydroxycinnamic acid while flavonoids are classified as flavones and their glycosides, catechins, flavonones, isoflavonoids, flavonols, anthocyanins, flavan-3-ols and proanthocyanidins flavonols, anthocyanins, flavan-3-ols, and proanthocyanidins. Tannins are either hydrolyzable or condensed [13,18].

Phenolics exist as free, soluble conjugates and in insoluble forms [19]. Conjugated soluble phenolics are bound to soluble low molecular mass molecules (carbohydrates, proteins, lipids) by either esterification at the carboxylic moiety or etherification at the hydroxyl group [20,21,22]. Insoluble phenolics are usually covalently bound to polymers such as polysaccharides and lignins through an ester linkage and are only released from the matrix through acid, alkaline, or enzyme hydrolysis [23,24]. In humans, insoluble phenolics are released from the matrix in the colon during the fermentation of the ingested material. The release of these phenolics has been identified as beneficial against colon cancer [25]. Free and conjugated phenolics are known as extractable phenolics while bound or insoluble phenolics are known as non-extractable [26]. Non-extractable or insoluble phenolics usually remain in the matrix of the residue after the extraction of soluble phenolics with aqueous alcohol [27,28]. Quantification of phenolics using only extractable phenolics results in the underestimation of the total phenolic content and antioxidant activity of the plant material [29]. It is therefore important to consider the contribution of the insoluble phenolics to the antioxidant activities of a plant material. 

*Terminalia sericea* Burch. ex DC. (Combretaceae) is a medicinal plant commonly found in the savannah woodlands of eastern, central, and southern Africa. [30]. The fruit, leaf, stem, or root have been used for the treatment of diabetes, diarrhea, venereal disease, and tuberculosis [31]. Decoction and infusion are common methods of herbal preparation for oral administration [32]. The Vhavenda people mix the root infusion in preparation of the baby’s soft porridge which helps in preventing diarrhea and dysentery [33,34]. Infusion or decoction prepared from *T. sericea* fruit, leaf, stem, or root is used to treat infectious wounds, diarrhea, eye infection, hypertension, fever, pneumonia, infertility, menorrhagia, stomach ache, cough, and gonorrhea [33,35]. Aqueous or organic extracts of *T. sericea* root, stem or leaf have been reported to possess antibacterial, antidiabetic, anti-HIV, anti-inflammatory, and anti-mycobacterial activities [31]. The crude extract of the root has been reported to be toxic to Monkey kidneys or Vero cells [36]. Previous studies [37,38,39] on the antioxidant activities of *T. sericea* have been limited to extractable antioxidants. Lupeol, inseparable mixtures of epicatechin-catechin and epigallocatechin-gallocatechin have been identified as antioxidant constituents of the acetone extract of stem bark [38]. There is, however, no available report on the estimation of the antioxidant activities of the non-extractable phenolics. It is therefore important to evaluate the antioxidant activities of the non-extractable phenolics to avoid underestimation of the antioxidant capacity. Several studies have been conducted on the quantification and antioxidant properties of extractable and non-extractable phenolics in fruits, vegetables, and cereals [40,41] compared to non-food medicinal plants. The aim of this study, therefore, is to evaluate the antioxidant activities of the extractable and non-extractable phenolics of *T. sericea*.

## 2. Results and Discussion

### 2.1. Extraction Yield

**Figure 1:**
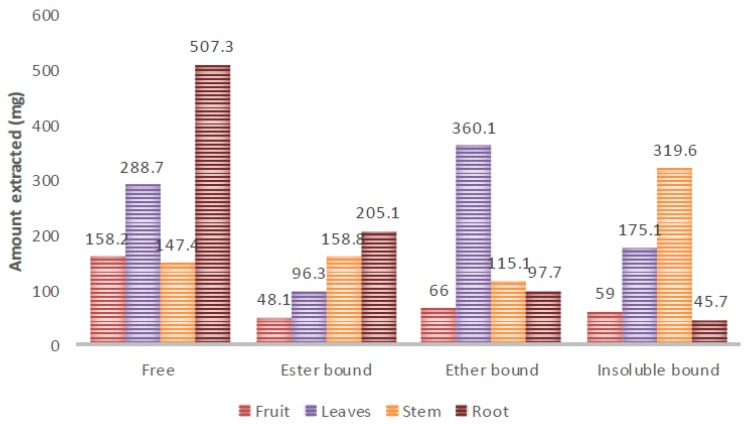
The extraction yield of extractable and non-extractable phenolics in the organs of *T. sericea.*

The result of the extraction yield is illustrated in Figure 1. Aqueous ethanol was used as the extracting solvent for extractable phenolic, so as to increase the extraction of very polar phenolics [42]. In general, the highest yield was found in the free phenolic root extract while the least yield was found in the insoluble phenolic root extract. Other studies have indicated that extractable (soluble) phenolics contain higher yields compared to non-extractable or bound phenolics [21,43], soluble or extractable phenolics have higher yields than bound or non-extractable phenolics. The root had the highest free and ester bound phenolic extract yield while the leaves had the highest ether bound phenolic extract yield and the stem had the highest insoluble bound phenolic extract yield. This result has indicated that there are more extractable phenolics in the root and leaf compared to the fruit and stem. However, the stem contained more non-extractable phenolics compared to the fruit, leaf, and root. While free phenolic acids contributed more to the extractable phenolics in the root, ether bound phenolics contributed more to the extractable phenolics in the leaf.

### 2.2. Total Phenolic Content

The result of the total phenolic content of the extractable and non-extractable phenolics is presented in Table 1. The highest TPC was found in the ester bound phenolic extract of the leaves (15.69 ± 0.04 mg GAE/g) and the root (15.62 ± 0.04 mg GAE/g) while the least TPC was found in the insoluble bound phenolic extract of the fruit (5.34 ± 0.02 mg GAE/g). In the fruit, the free and ether bound phenolics displayed the highest TPC, while the ester bound phenolics contained the highest TPC in the leaves. The free and ester bound phenolics contained the highest TPC in both stem and root. Considering the distribution of the TPC in the organs, the stem and the root displayed the highest free phenolics while the root and leaves displayed the highest ester bound phenolics. 

In the ether bound phenolics, the fruit and stem contained the highest total phenolic content while the leaves and the stem displayed the highest total phenolic content in the insoluble bound phenolics. The ester bound phenolics contained the highest average TPC (15.11 mgGAE/g) while the least average TPC was found in the insoluble bound (9.74 mgGAE/g). Although the stem contained the highest TPC (14 mgGAE/g), the actual difference between the TPC of the organs was 1–2 mgGAE/g. This study has demonstrated that the total phenolic content in the extractable phenolics was higher than non-extractable phenolics. Furthermore, the stem contained the highest average total phenolic content (14 mgGAE/g). It also demonstrated that free and ester bound phenolics contained more total phenolic content compared to the ether and insoluble bound phenolics. This study correlates to the study done by Nayaka et al. [44], which indicated that swallow root free phenolics contained higher phenolic compounds compared to the insoluble phenolic extract. The study by Kumar et al. [45] also indicated higher free phenolic content than the bound phenolics in *Emlica officinalis* and *Curcuma longa*. Gallic acid and tannic acid were found to be the major phenolics in both free and bound phenolics of *E. officinalis.* Curcumin was the major phenolic found in the free phenolics of *C. longa* while proto-catechuic acid and ferulic acid were the main phenolics in the bound phenolics. However, the study by Singh et al. [21] indicated that insoluble phenolics contained more total phenolic content in *Moringa oleifera* seed flour compared to the soluble phenolics. Gallic acid was found to be the main phenolic in the soluble phenolics while catechin and epicatechin were found to be the major phenolics in the insoluble phenolics in *M. oleifera*. In another study [46], the TPC of non-extractable phenolics was higher than the extractable phenolics of Cashew apple. The non-extractable phenolic of Mandrin waste has been reported to contain higher TPC compared to the extractable phenolics [47].

### 2.3. Antioxidant Activities

The result of the antioxidant activities of the soluble and insoluble extracts is presented in Table 2. The free and insoluble phenolics of the fruit displayed the highest (3.13 ± 0.75 µg/mL) and the lowest (235 ± 6.69 µg/mL) antioxidant activities, respectively. In the fruit, the free phenolics displayed the highest antioxidant activity while the ester bound phenolics (4.58 ± 0.71 µg/mL) displayed the highest antioxidant activity in the leaves. The free phenolics (8.78 ± 0.57 µg/mL) displayed the highest antioxidant activity in the stem but was not significantly different (*p* > 0.05) from the ester bound phenolics (9.32 ± 0.42 µg/mL). In the root, the ester bound phenolics displayed the highest antioxidant activities (4.89 ± 0.34 µg/mL). Considering the distribution of the antioxidants in the organs, the free phenolics of the fruit and leaves displayed higher antioxidant activity (*p* < 0.05) compared to the stem and root. The ester bound phenolics of the fruit, leaves, and root displayed higher antioxidant activities (*p* < 0.05) compared to the stem. In the glycoside or ether bound phenolics, the stem displayed the highest antioxidant while the leaves displayed the highest antioxidant activity in the insoluble phenolics. The ester bound phenolics displayed the best antioxidant activity with an average IC_50_ value of 6.4 µg/mL while the stem displayed the best antioxidant activity with the IC_50_ value of 13.2 µg/mL. 

This study has indicated that extractable phenolics, especially free and ester bound phenolics, possess higher antioxidant activities compared to the insoluble phenolics. This study also demonstrated that the stem contained more antioxidant compounds compared to the fruit, leaves, and root. In a previous study [39], the crude extracts of *T. sericea* stem displayed higher antioxidant activity compared to the leaves and the root. The ester bound extractable phenolics displayed the highest average antioxidant activity compared to the free, glycoside bound extractable phenolics and non-extractable (insoluble) phenolics. In general, the extractable phenolics (free, ester, and glycoside) displayed higher antioxidant activities compared to the non-extractable phenolics. The higher antioxidant activity in the extractable phenolics could be due to the higher total phenolics compared to the non-extractible (insoluble) phenolics. This result corresponds to the findings of Nayaka et al. [44], in which the conjugated phenolics of *Decalepis hamiltonii* root displayed higher antioxidant activities compared to the insoluble phenolic extract. However, other studies have indicated that insoluble bound phenolics display higher antioxidant activities in fruits, vegetables, and cereals [19]. There is no available literature of any report of antioxidant activities of extractable and non-extractable phenolics from genus *Terminalia.*

### 2.4. Multivariate Analysis

#### 2.4.1. Agglomerative Hierarchical Clustering (AHC) Analysis 

**Figure 2:**
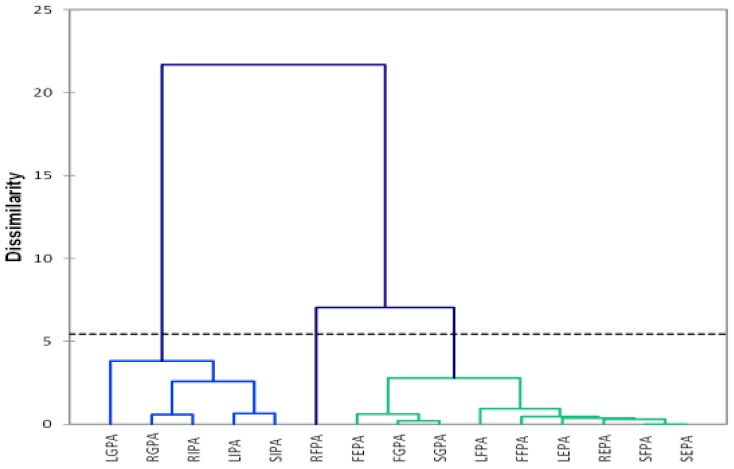
The dendrogram of extractable and non-extractable phenolics in the fruit, leaves, stem, and root of *T. sericea.*

Agglomerative hierarchical clustering is a multivariate analytical tool used to cluster samples based on dissimilar characteristics and then displayed as a dendrogram [48,49]. The dendrogram (Figure 2) was constructed by Euclidean Pythagorean distance dissimilarities with Ward’s Method of linkage [50,51]. Dotted lines represent automatic (entropy) truncation [49].

In this study, extractable phenolics include free, ester bound, and ether or glycoside bound phenolic acids while non-extractable phenolics are also known as insoluble phenolic acids. Ether bound extractable phenolics are also designated glycoside phenolics.

The dendrogram constructed indicated three clusters, indicating the dissimilarities in the extractable and non-extractable phenolics in the fruit, leaf, stem, and root of *T. sericea*. Cluster 1 consists of Leaf insoluble phenolic acid (LIPA), stem insoluble phenolic acid (SIPA), root insoluble phenolic acid (RIPA), leaf glycoside phenolic acid (LGPA), and root glycoside phenolic acid (RGPA). The second cluster consists only of root free phenolic acids (RFPA). The third cluster consists of fruit ester phenolic acid (FEPA), leaf ester phenolic acid (LEPA), stem ester phenolic acid (SEPA), root ester phenolic acid (REPA), fruit free phenolic acid (FFPA), leaf free phenolic acid (LFPA), stem free phenolic acid (SFPA), fruit glycoside phenolic acid (FGPA), and stem glycoside phenolic acid (SGPA). The sample FIPA (fruit insoluble phenolic acid) was removed from the plot due to the high IC_50_ value (235 μg/mL), making it an outlier compared to the values of the other samples. The dendogram clearly indicates that the glycoside bound extractable and non-extractable (insoluble) phenolics are similar and different from the phenolics found in the free and ester bound phenolic extracts. The widest variety was observed between LGPA in cluster 1 and SEPA in cluster 3. Although FGPA and SGPA are found in cluster 3, they are also found in the same sub-cluster, indicating similarities in their phenolics. The clustering of RFPA alone in cluster 2 may be due to the high extract yield and total phenolic content. This result has indicated similarities between glycoside bound extractables and non-extractable phenolics, and dissimilarities with free and ester bound extractable phenolics.

#### 2.4.2. Principal Component Analysis

**Figure 3:**
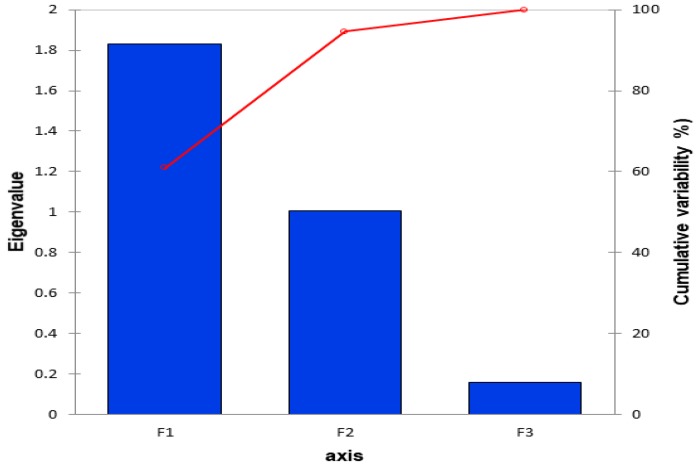
The scree plot indicating the number of significant factors explaining the variations. F1–3: factors 1–3.

**Figure 4:**
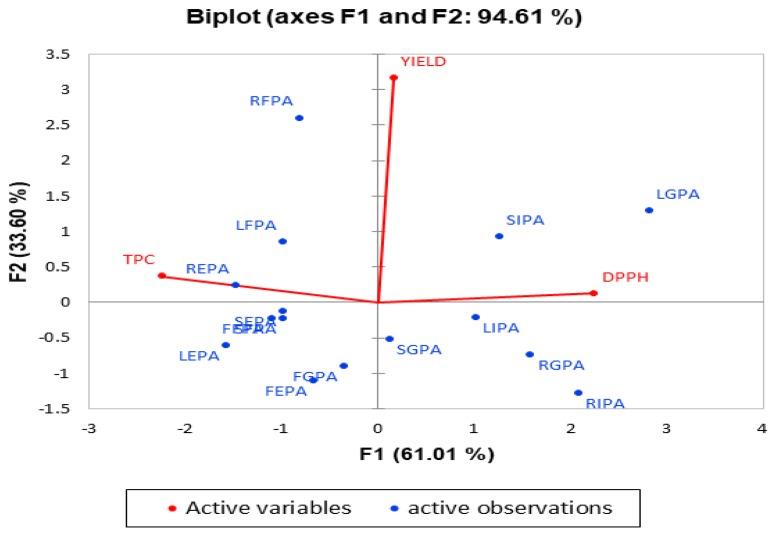
The principal component analysis biplot indicating the correlation circle of the active variables and the scatter plot of the active observations. F1: the first factor or principal component 1 (PC1). F2: the second factor or principal component 2 (PC2).

The Principal Component Analysis is an unsupervised method used to identify the patterns and grouping within a data set based on the highest variation within the data [52]. The scree plot (Figure 3) presented 3 factors, however, only F1 and F2 were significant to explain 96% of the variations. A biplot illustrating the PCA correlation and score scatter plot is presented in Figure 4. 

There was no correlation (Table 3) between extraction yield and TPC (r = 0.034), and between extraction yield and DPPH (r = 0.097). However, there was a strong negative correlation between TPC and DPPH (r = −0.828). 

About 61% of the variation in the samples was explained by factor 1 (principal component PC1) while 34% of the variation was explained by factor 2. The scatter plot revealed that the insoluble phenolics and glycosides are found in positive F1 (cluster 1) free and ester bound phenolics are found in the negative F1 (cluster 2). The samples found along the positive F1 have high IC_50_ indicating low antioxidant activities. Although FGPA is found in negative F1, it is closer to the center. This further suggests that glycosides have lower antioxidant activities. This indicates that glycosides and insoluble phenolics are poor antioxidants. The result of the antioxidant activities (Table 2) indicated that all the insoluble and glycoside (ether bound) phenolics had higher IC_50_ values (lower antioxidant activity) compared to the free and ester bound phenolic extracts. 

Along negative F1, REPA, and LEPA were the farthest samples from the center. The highest total phenolic contents (Table 1) were found in both samples. Free and ester bound phenolics contained higher phenolics and exhibited higher antioxidant activities compared to the glycosides and the insoluble phenolics. This trend explains the negative relationship between TPC and DPPH (r = −0.828). Previous studies have reported on the correlation between TPC and DPPH antioxidant activities [53,54]. The variation in the extraction yield is explained along F2. Positive F2 consists of RFPA, LFPA, SIPA, LGPA, and REPA. The highest extractable phenolics were found in leaves and root while the highest non-extractable phenolics were found in the stem. Although LGPA had a high extraction yield, it displayed a poor antioxidant activity. The same trend was found with SIPA. On the other hand, the extraction yield and antioxidant activity (low IC_50_ values) of RFPA, REPA, and LFPA were high. This trend explains why there was no correlation between DPPH and extraction yield. Although FIPA was not included in the constructing the PCA model, it contained the least phenolic content and antioxidant activity.

### 2.5. Nuclear Magnetic Resonance (NMR) Spectroscopy

**Figure 5:**
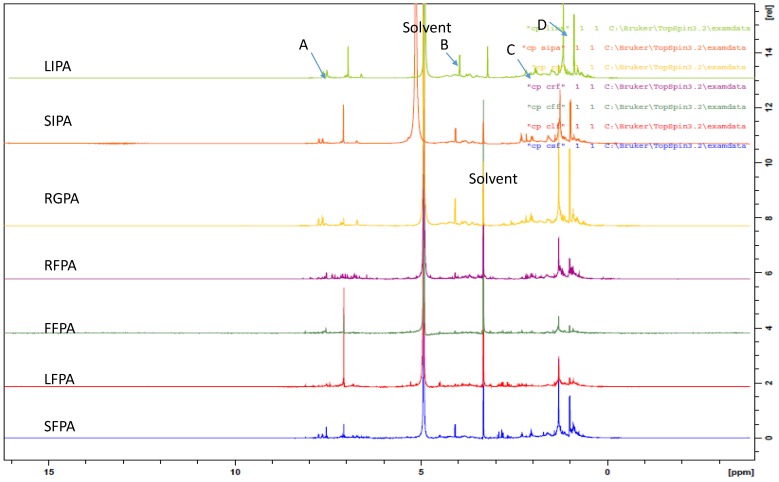
The ^1^H-NMR spectra indicating the chemical shifts identical to Pthalates.

**Figure 6:**
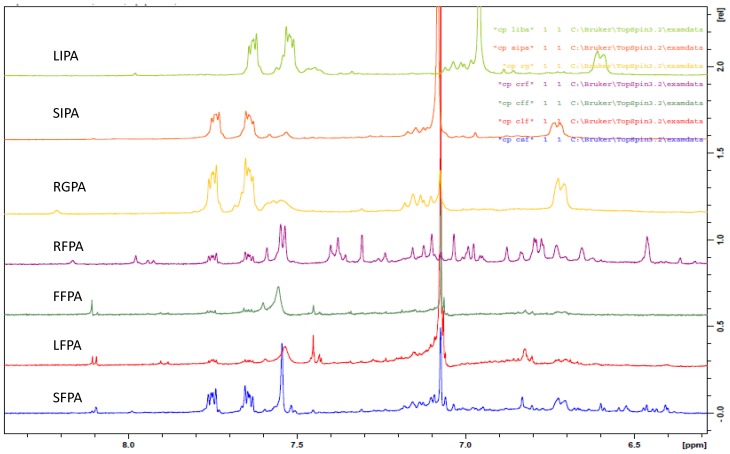
The ^1^H-NMR spectra indicating the chemical shift (δ ppm) of the phenolic acid extracts at the aromatic region.

The advantage of NMR metabolomics is its ability to detect a wider range of metabolites compared to GC/MS and LC/MS. It is also highly reproducible and non-destructive [55,56]. NMR was used in this study to identify the variation in metabolites responsible for the activity and grouping in the multivariate analysis. The results of the one-dimensional proton NMR analysis of the extractable and non-extractable phenolics are presented in Figure 5 and Figure 6. The samples were selected for the NMR analysis based on their clustering in the AHC and PCA analysis. In the two statistical tools, free and ester bound phenolics were clustered in group 2 (or 2 and 3 in the case of AHC) while the glycosides and insoluble phenolics were clustered in group 1. All the free phenolics were selected to represent the cluster 2 (or 2 and 3, in the case of AHC) while LIPA, RGPA, and SIPA were selected to represent cluster 1. Multiplet signals (Figure 5) at aromatic region δ_H_ 7.7 and 7.5 ppm (A), δ_H_ 4.2 ppm (B), δ_H_ 1.7–1.27 ppm (C), and δ_H_ 0.92 ppm (D) are characteristics of phthalates, which are contaminants from the solvents used for the extraction [57]. The solvent peaks are indicated at δ_H_ 5 ppm and δ_H_ 3.2 ppm. 

In the proton NMR spectra, δ_H_ 0.5–3.0 ppm are the aliphatic or organic, amino acid region while the chemical shift ranges δ_H_ 3.0–5.5 ppm and 5.5–10 ppm are predominantly the carbohydrate and aromatic regions, respectively [58,59]. Signals with chemical shifts characteristics of carbohydrates were not visible in any of the spectra. The result of the aromatic region of the extractable and non-extractable phenolics is presented in Figure 6.

The fruit free phenolic acid (FFPA) displayed four singlet signals at δ_H_ 8.11, 7.60, 7.45, and 7.07 ppm. The leaf free phenolic acid (LFPA) displayed a doublet signal at δ_H_ 8.10 ppm (*J* = 4.8 Hz), two singlet signals at δ_H_ 7.54 and 7.07 ppm, and two doublets at δ_H_ 7.43 (*J* = 2 Hz) and 6.81 (*J* = 8.8 Hz). The stem free phenolic acid (SFPA) displayed singlet signals at δ_H_ 8.09, 7.54, 7.07, 6.83 ppm and a doublet signal at δ_H_ 6.72 ppm (*J* = 9.6 Hz). The root free phenolic acid (RFPA) displayed seventeen singlet signals at δ_H_ 8.17, 7.98, 7.59, 7.31, 7.24, 7.16, 7.12, 7.10, 7.07, 7.03, 6.88, 6.84, 6.73, 6.65, 6.46, 6.36, and 6.32 ppm and six doublets signals at δ_H_ 7.94 ppm (*J* = 8.0 Hz), 7.54 ppm (*J* = 5.2 Hz), 7.39 ppm (*J* = 8.8 Hz), 6.98 ppm (*J* = 6.0 Hz), 6.78 ppm (*J* = 8.8 Hz), and 6.19 ppm (*J* = 12 Hz). The glycoside (ether) bound phenolic acid (RGPA) displayed two singlet signals at δ_H_ 8.21, 7.08 ppm and one doublet signal at δ_H_ 6.72 ppm (*J* = 6.8 Hz). The leave insoluble phenolic acid (LIPA) displayed a singlet signal at δ_H_ 6.96 ppm and a doublet signal at δ_H_ 6.6 ppm (*J* = 8.8 Hz). The stem insoluble phenolic acid (SIPA) displayed a singlet signal at δ_H_ 7.08 ppm and a doublet signal at δ_H_ 6.73 ppm (*J* = 7.2 Hz). 

All the extractable phenolics (FFPA, LFPA, SFPA, RFPA, and RGPA) displayed a singlet signal around δ_H_ 8.0 ppm. This signal was not found in the insoluble phenolics (LIPA and SIPA). This could be a major reason for the clear separation in the three classes illustrated in the dendrogram (Figure 2) and the scatter plot (Figure 3). Additionally, included in the PCA score plot class two with the insoluble phenolics were the glycosides (SGPA, RGPA, and LGPA). The clustering of RGPA in cluster 1 may be due to the absence of the singlet signal range δ_H_ 7.60–7.31 ppm compared to the other extractable phenolics. The absence of these signals could be responsible for the poor antioxidant activity of all the extracts found in cluster 1 of the PCA score plot. 

Comparing the spectra within the extractable phenolics, LFPA was the only extract with a doublet around δ_H_ 8.0 ppm. This may be a distinguishing feature of the leaf compared to the other organs. Another unique feature in the leaf is the similarities in the doublet signals around δ_H_ 6 ppm (*J* = 8.8 Hz) in both LFPA and LIPA. The RFPA displayed the highest number of signals (Figure 6), indicating the presence of more phenolics compared to other extracts. The highest extract yield found in RFPA (Figure 1) could be due to the presence of more phenolics as indicated by the ^1^H-NMR spectra. All the extracts, except LIPA, displayed a singlet at δ_H_ 7.07 ppm. This chemical shift is consistent with the characteristic signal for gallic acid, a hydroxybenzoic acid [60,61,62]. Doublets at δ_H_ 7.94 ppm (*J* = 8 Hz) and δ_H_ 7.39 ppm (*J* = 8.8) in the RFPA spectra are characteristic signals for hydroxybenzoic acid derivatives [63]. The singlet signal range δ_H_ 6.73–6. 32 ppm in the RFPA spectra is consistent with the signals of the aromatic rings of resveratrol-3-*O*-β-rutinoside, a hydroxystilbene glycoside [64], which has been previously isolated from the root of *T. sericea* [65,66]. The chemical shifts in the spectra of the selected samples suggest that hydroxybenzoic acid and stilbenes are the major phenolics of *T. sericea*. 

**Figure 7:**
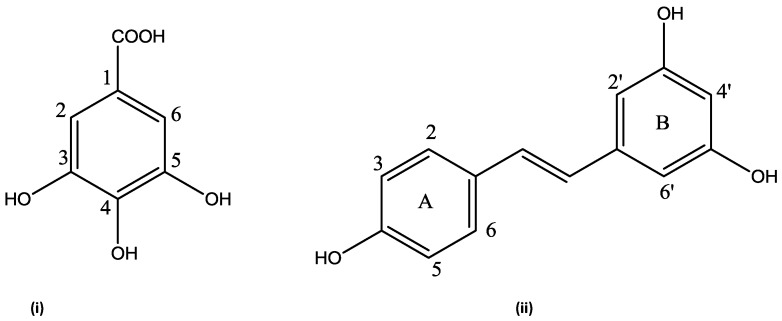
The structure of identified compounds (**i**) Gallic acid (**ii**) resveratrol from RFPA.

The presence of gallic acid (Figure 7) in RFPA was confirmed by comparing the 1D and 2D spectra of the gallic acid standard and RFPA. The cross peak between δ_H_ 7.07 ppm and δ_C_ 108.9 ppm in the heteronuclear single quantum correlation (HSQC) of the gallic acid (Supplementary Figure S1) corresponds with the cross peak in RFPA (Supplementary Figure S2). The proton δ_H_ 7.07 ppm was identified as the methine proton of C-2 or 6 (δ_C_ 108.9 ppm) in gallic acid. The carbon δ_C_ 139.6 ppm (Supplementary Figure S2) was assigned to C-3 or 5 while carbon δ_C_ 145.0 ppm was assigned to C-4 [63]. The chemical shifts δ_C_ 120.6 ppm and δ_C_ 169.0 ppm were assigned to C-1 and the carbonyl functional group (C = O), respectively. The cross peaks between δ_C_ 108.9 ppm and singlet protons δ_H_ 7.03, 7.10, 7.12, and 7.16 ppm suggest the presence of gallic acid derivatives or other hydroxybenzoic acid derivatives. In the heteronuclear multiple bond correlation (HMBC) spectrum of RFPA (Supplementary Figure S3), the cross peaks of δ_H_ 7.03 and 7.07 with δ_C_ 139.6 ppm further confirm that δ_H_ 7.03 ppm is characteristics of a hydroxybenxoic acid, probably a derivative of gallic acid. Gallic acid has been isolated from the fruit of *T. bellerica* [67], the fruit pulp of *T. chebula* [68], and the leaf of *T. arjuna* [69]. However, this is the first report of gallic acid in *T. sericea*. This study has demonstrated that gallic acid is a major phenolic compound in *T. sericea*. According to Ajila and Prasada Rao [70], gallic acid was a major phenolic acid in both raw and ripe badami and raspusi mango peel. Gallic acid and its derivative (gallic hexoside) were the major phenolic acid in berry seed meals [71].

**Figure 8:**
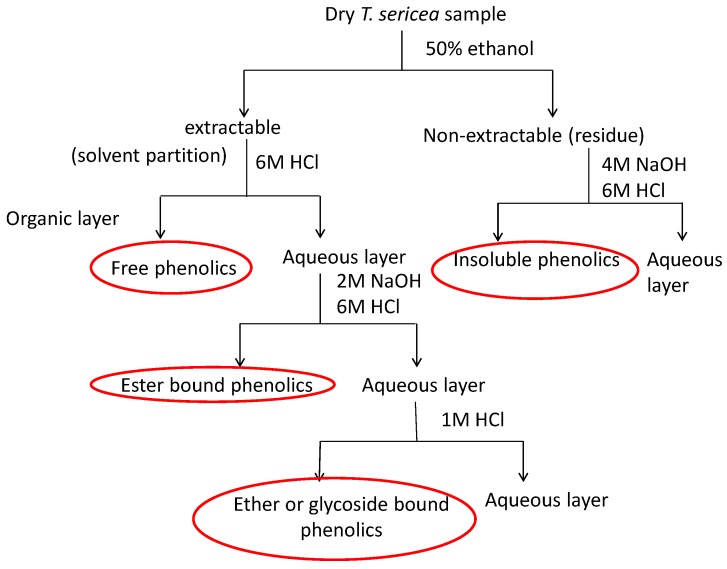
The flowchart indicating the extraction of free, ester, ether, and insoluble-bound phenolics.

Resveratrol consists of two aromatic rings (A and B) linked by an olefin (Figure 8). The A ring is characterized by two doublets while the B ring is characterized by two singlets. In the HSQC spectrum (Supplementary Figure S2) of RFPA, the cross peak between the doublet at δ_H_ 7.39 ppm (*J* = 8.4 Hz) and δ_C_ 128.5 ppm corresponds to the carbons C-2 or C-6 in the A ring. The cross peak between the doublet at δ_H_ 6.79 ppm (*J* = 8.8 Hz) and δ_C_ 115.1 ppm corresponds to the carbons C-3 or C-5. The identification of these cross peaks in RFPA confirms the A ring. In the HMBC spectrum (Supplementary Figure S3) of RFPA, the cross peaks of δ_H_ 7.39 and 6.79 with δ_C_ 128.5 further confirmed that both doublet protons are in ring A. Both doublets also displayed cross peak with δ_C_ 158.1 ppm. In the B ring, the cross peak between the singlet proton δ_H_ 6.73 ppm and δ_C_ 106 ppm corresponds to the carbon C-2′ or C-6′. 

In the ^13^C spectrum (Supplementary Figure S4), the chemical shift δ_C_ 102.5 ppm was assigned to C-4′ due to the cross peak between δ_C_ 102.5 ppm and the singlet proton δ_H_ 6.46 ppm in the HSQC. The chemical shift δ_C_ 160.1 ppm was assigned to C-3′ or C-5′ while δ_C_ 158.1 ppm was assigned to C-4 in the A ring [64] due to the cross peak between both δ_H_ 7.39 and 6.79 and δ_C_ 158.1 ppm in the HMBC spectrum. 

The NMR data (one and two-dimensional) have revealed that gallic acid and resveratrol are major phenolics in the root free phenolic acid. It also revealed that gallic acid is present in both extractable and non-extractable extracts of all organs. In other studies [21,43,72,73], gallic acid has been reported to be present in both extractable and non-extractable phenolics. The absence of the chemical shifts δ_H_ 7.39, 6.79, 6.73, and 6.65 ppm in the fruit, leaf, and stem suggests that resveratrol is not present or in very little amount.

## 3. Materials and Methods

### 3.1. Materials

Solvents were purchased at Rochelle Chemicals, South Africa, while the reagents were purchased from Merck (Darmstadt, Germany). The fruit, leaf, stem, bark, and root samples of *T. sericea* were collected from Vuwani, Limpopo province in June 2014 and identified by Prof Tshisikhawe MP (Department of Botany, University of Venda). The collected voucher specimen (MPT00114) was identified and deposited at the University of Venda Herbarium.

### 3.2. Extraction

The samples were washed to remove debris and air dried for two weeks. The dried materials were ground to a powder using an industrial grinder (Dietz-motoren KG, Dettingen unter Teck, Germany). The free, conjugated and bound phenolics were extracted as described by Chandrasekara and Shahidi [20]. Four grams of each dried sample was macerated with 40 mL of 50% ethanol for 24 h at room temperature. The supernatant was filtered and the residue was re-extracted with the same volume of the extracting solvent on an orbital shaker for 1 h. This process was repeated three times and all the filtrate were combined to obtain extractable or soluble phenolics. The leaf and fruit samples were previously defatted with hexane to remove the fat content before extraction [21]. The summary of the extraction of the extractable and non-extractable phenolics is illustrated in Figure 8.

#### 3.2.1. Free Phenolics

The soluble phenolic aqueous solution was acidified with HCl (6 M; pH 2) and was partitioned with diethyl ether:ethyl acetate (1:1) in a separating funnel. The organic layer containing the free phenolics was collected. The aqueous layer was further partitioned (4×) with a mixture of diethyl ether and ethyl acetate to extract the free phenolics. The organic layers were combined and evaporated to dryness using a rotar vapour (Buchi, Flawil, Switzerland). 

#### 3.2.2. Ester Bound Phenolics

The aqueous fraction (10 mL) obtained after the extraction of the free phenolics was hydrolysed with 40 mL of 2 M NaOH for 4 h at room temperature. The alkaline solution was made acidic with 6 M HCl and the ester bound phenolics were extracted with diethyl ether: the ethyl acetate mixture as described previously.

#### 3.2.3. Glycoside Bound Phenolics

The aqueous fraction (20 mL) obtained after the extraction of the ester bound phenolics was subjected to acid hydrolysis with HCl (1 M; 30 mL) by incubating in a water bath at 95 °C for 45 min. The glycoside bound phenolics were extracted with the organic mixture and dried as described earlier.

#### 3.2.4. Insoluble Phenolics

The residue after the extraction of the soluble phenolics was hydrolysed with NaOH (4 M; 40 mL) for 1 h at room temperature. The solution was acidified with HCl (6 M; pH 2) and the insoluble phenolics were extracted with the organic mixture and dried as described earlier.

### 3.3. Total Phenolic Content

The total phenolic contents of the free, conjugated and insoluble phenolic extracts were determined using a method described by Anokwuru et al. [74]. Briefly, the extracts (1 mg/mL; 20 μL) were transferred to 96 well plates containing 80 μL of distilled water. Follin Ciocalteu reagent (10%; 20 μL) was added to each well containing the diluted extracts. The mixture was allowed to stand for 1 min at room temperature before Na_2_CO_3_ (7%; 60 μL) was added to each well. The mixture was further diluted with 120 μL of distilled water and incubated for 30 min at room temperature before measuring the absorbance with a microplate reader (Versa Max, Shanghai, China) at 760 nm. The total phenolic content was extrapolated from a gallic acid (10–80 μg/mL) calibration curve (y = 0.0009x + 0.049; R^2^ = 1) and was expressed as mg Gallic Acid Equivalent per gram of the dry extract (mgGAE/g) using Equation (1) [68].

C = cV/m (1)

C = the total phenolic content (mgGAE/g)
c = the concentration of Gallic acid obtained from the calibration curve
V = the volume of extract (mL)
m = the mass of the extract (g)

### 3.4. Antioxidant Activity

The antioxidant activity was determined using a DPPH (2,2-diphenyl-1-picrylhydrazyl) assay as described by Anokwuru et al. [74]. Briefly, 100 μL of the extracts (1 mg/mL) or gallic acid (0.10 mg/mL;) were serially diluted in a 96 well plate and DPPH solution (0.3 mM; 200 μL) was added to each well containing the diluted samples. The mixtures were left in the dark at room temperature for 30 min and the absorbance was read at 517 nm using a microplate reader (Versa Max, Shanghai, China). The blank contained only distilled water and the DPPH solution without any sample or gallic acid.

The following equation was used to calculate the percentage antioxidant activity (AA)

%AA = [Ab − As/Ab] × 100 (2)

where Ab is the absorbance of the blank and As is the absorbance of the sample or gallic acid. The concentration required for 50% inhibition (IC_50_) of the DPPH free radical [75,76] was derived from a plot of % AA against concentration. 

### 3.5. Nuclear Magnetic Resonance (NMR) Spectroscopy

The proton spectra of selected extractable and non-extractable phenolic extracts were recorded on Bruker Ultra Shield^TM^ Plus 400 MHZ (Biospin) (Bruker, Bellericea, MA, USA). The obtained spectra were processed using Bruker Topspin 3.2 on the AVIII 400 software. The extracts (10 mg) were dissolved in 1 mL deuterated methanol (Methanol-*d*4) and 700 μL was transferred into an NMR tube for analysis. Gallic acid and resveratrol were identified in the crude extract by comparing the 2D experiment (heteronuclear single quantum correlation, HSQC; heteronuclear multiple bond correlation, HMBC) of gallic acid standard and resveratrol-3-*O*-β-rutinoside (previously isolated).

### 3.6. Statistical Analysis

The total phenolic content and antioxidant activity were analysed in triplicates and the values were expressed as the mean ± standard error. A one-way ANOVA was used to determine the significant difference in the TPC and the antioxidant activities using SPSS 23. Fisher’s least significant difference (LSD) method was used for post hoc analysis. Multivariate analysis (Agglomerative hierarchical clustering and principal component analysis) were performed on the data obtained (percentage yield, total phenolic content, and antioxidant activity) using the software XLSTAT (2017). Agglomerative hierarchical clustering (AHC) analysis was used to determine the dissimilarities between the extractable and non-extractable phenolics [48]. Principal component analysis used was Pearson correlation [26]. The scree plot was used to determine the number of significant PCA factor [77]. 

## 4. Conclusions

This study demonstrated that free and ester bound extractable phenolics contained higher total phenolic content and antioxidant activity compared to the ether bound or glycoside and insoluble phenolics of *T. sericea*. Furthermore, the use of aqueous alcohol does not underestimate the amount of phenolic compounds and antioxidant in the organs. The dendrogram constructed from the agglomerative hierarchical clustering (AHC) indicated three clusters based on the type of phenolics extracted. The insoluble and glycosides were found in cluster 1 while the free and ester bound extractable phenolics were found in cluster 3. Cluster 2 contained free phenolic acids from the root alone. The three clusters from the dendrogram were further confirmed with the principal component analysis (PCA) score plot. There was a significant (*p* < 0.05) correlation between the total phenolic acid and the DPPH antioxidant activity. One-dimensional ^1^H-NMR spectra of the selected extracts revealed that hydroxybenzoic acids and stilbenes are major phenolics in the plant. The difference in the antioxidant activity of cluster 1 compared to cluster 2 (or 2 and 3 in the case of AHC) could be due to the variation in the chemical shift between δ_H_ 8.1 and 7.07 ppm. In the 2D experiment, the heteronuclear single quantum correlation (HSQC) and heteronuclear multiple bond correlation (HMBC) of RFPA revealed that Gallic acid and resveratrol are major phenolic constituents of *T. sericea* root. Further study is required for the identification of phenolics responsible for the antioxidant activities in *T. sericea*.

## Figures and Tables

**Table 1:** The total phenolic contents (mg GAE/g) of extractable and non-extractable pehnolics in *T. sericea*.

Organs	Free	Ester Bound	Ether Bound	Insoluble Bound	Average
Fruit	14.30 ± 0.02 ^a^	13.87 ± 0.13 ^c^	14.25 ± 0.05 ^a^	5.34 ± 0.02 ^g^	11.94
Leaves	14.81 ± 0.13 ^a^	15.69 ± 0.04 ^b^	11.17 ± 0.12 ^e^	11.60 ± 0.12 ^e^	13.32
Stem	15.12 ± 0.01 ^b^	15.24 ± 0.05 ^b^	14.03 ± 0.08 ^a^	11.62 ± 0.02 ^e^	14.00
Root	15.12 ± 0.07 ^b^	15.62 ± 0.04 ^b^	12.12 ± 0.14 ^d^	10.38 ± 0.04 ^f^	13.31
Average	14.84	15.11	12.89	9.74	

The values in each column or row with different alphabetical letters are significantly different at *p* < 0.05.

**Table 2:** The antioxidant activity (IC_50_ µg/mL) of free and bound phenolics in the organs of *T. sericea*.

Organs	Free	Ester Bound	Glycoside Bound	Insoluble Bound	Average
Fruit	3.13 ± 0.75 ^a^	6.91 ± 0.75 ^a^	12.6 ± 0.2 ^c^	235 ± 7 ^g^	64.4
Leaves	6.44 ± 0.81 ^a^	4.58 ± 0.71 ^a^	34.2 ± 1.6 ^d^	15.4 ± 1.3 ^e^	15.2
Stem	8.78 ± 0.57 ^b^	9.32 ± 0.42 ^b^	17 ± 0.7 ^e^	17.8 ± 0.8 ^e^	13.2
Root	8.99 ± 0.53 ^b^	4.89 ± 0.34 ^a^	25.3 ± 0.6 ^f^	23.1 ± 0.5 ^f^	15.6
Average	6.8	6.4	22.2	72.8	
Gallic acid					5.5 ± 0.1

The values in each column or row with different alphabetical letters are significantly different at *p* < 0.05.

**Table 3:** The correlation analysis between parameters and *T. sericea* phenolic acid extracts.

Parameters	YIELD	TPC	DPPH
YIELD	1	0.034	0.097
TPC	0.034	1	−0.828
DPPH	0.097	−0.828	1

TPC: total phenolic content; DPPH: 2,2-diphenyl-1-picrylhydrazyl.

## Supplementary Materials

Supplementary materials are available online.
