# Supplementary material for: Antioxidant Activity and Spectroscopic Characteristics of Extractable and Non-Extractable Phenolics from Terminalia sericea Burch. ex DC
Source: Molecules. 2018 May 29;23(6):1303. doi: 10.3390/molecules23061303 (PMC6099621; doi:10.3390/molecules23061303)
Supplement: Supplementary file 1 [file molecules-23-01303-s001.pdf]

SUPPLEMENTARY FIGURE

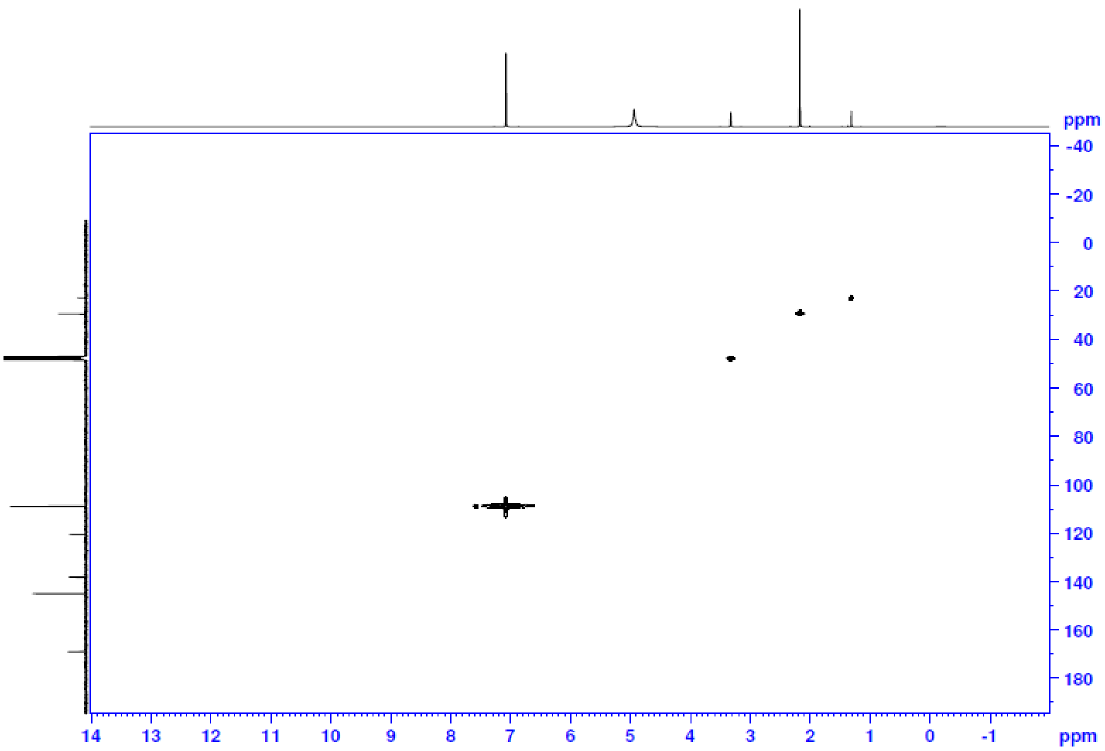

Supplementary Figure 1: HSQC spectrum of gallic acid standard

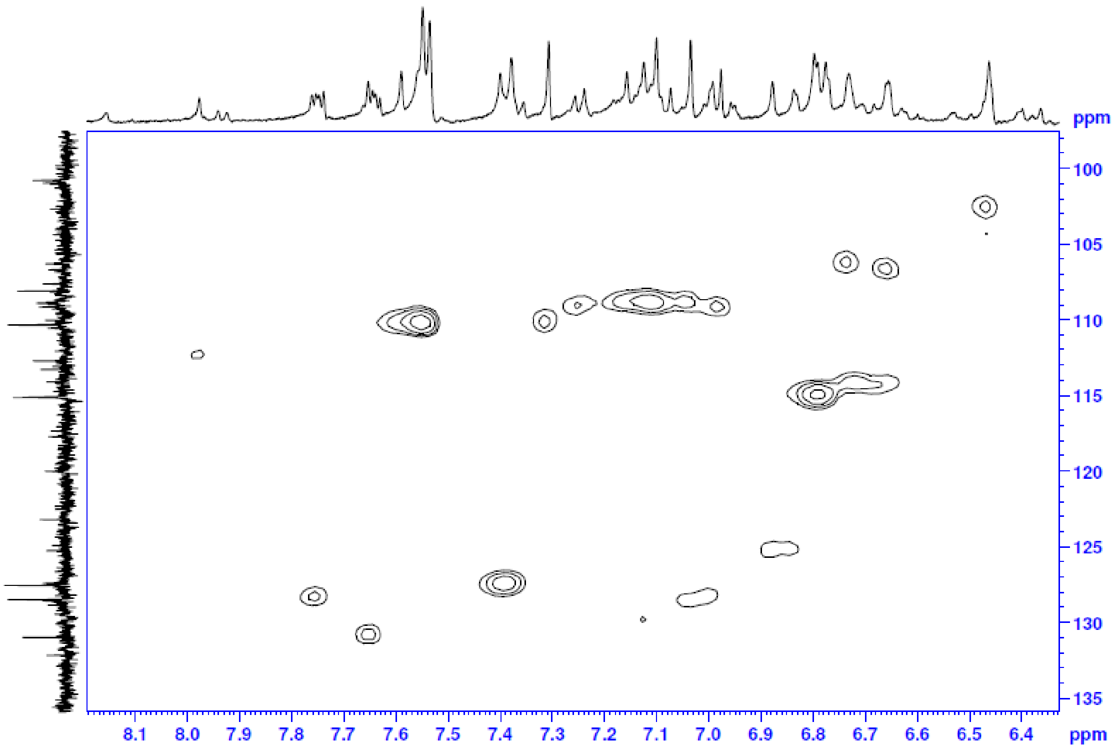

Supplementary Figure 2: HSQC spectrum of RFPA

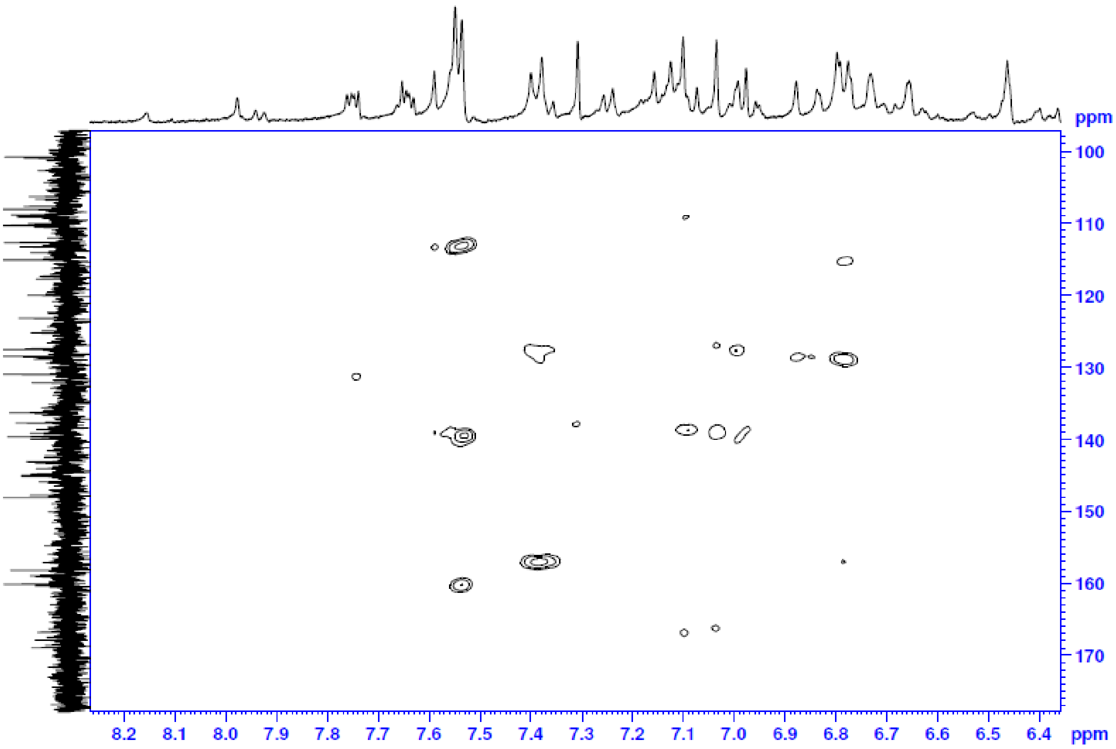

Supplementary Figure 3: HMBC spectrum of RFPA

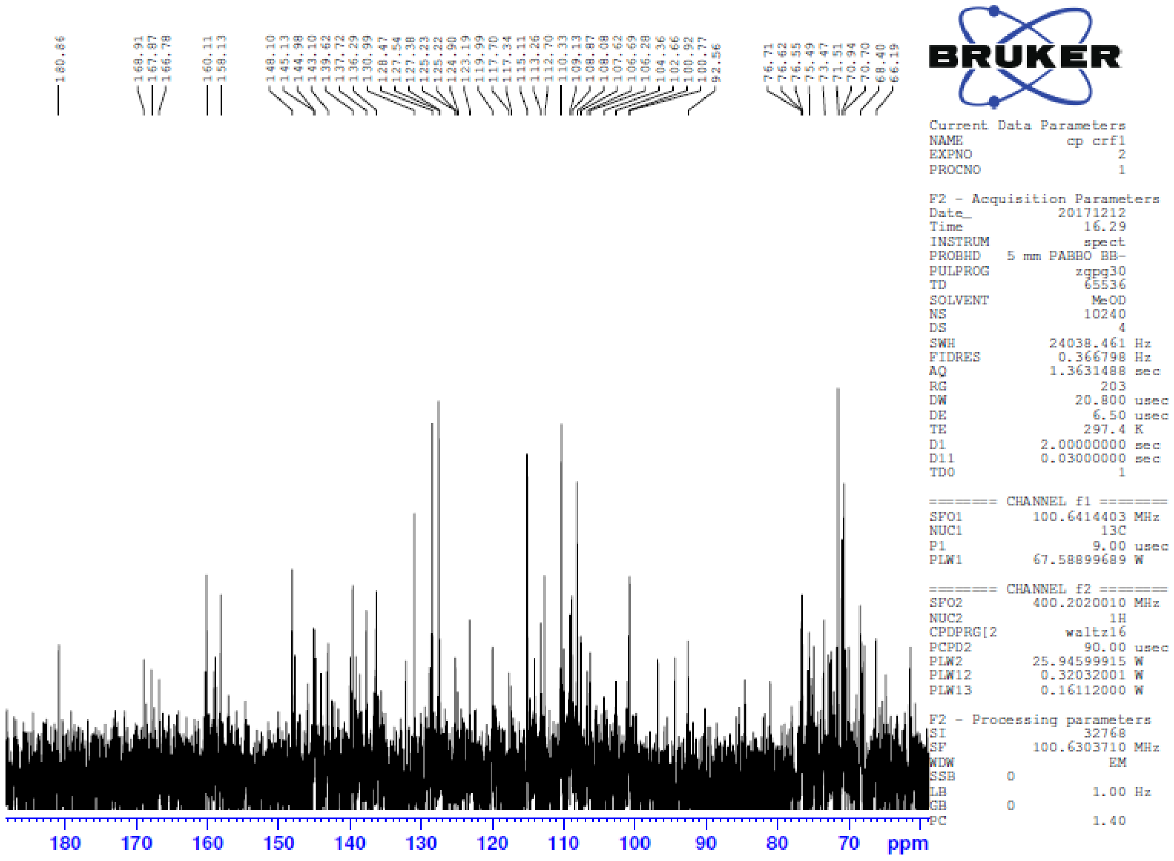

Supplementary Figure 4: ^{13}C spectrum of RFPA
